# Enhanced extracellular respiration of engineered *Bacillus subtilis* via anodic electro-fermentation with pH optimisation

**DOI:** 10.1186/s13068-025-02731-5

**Published:** 2026-01-03

**Authors:** Yu Sun, Changshuo Liu, Igor Vassilev, Antti J. Rissanen, Jin Luo, Marika Kokko

**Affiliations:** 1 Faculty of Engineering and Natural Sciences, Tampere University, Korkeakoulunkatu 8, 33720 Tampere, Finland; 2 Natural Resources Institute Finland, Latokartanonkaari 9, 00790 Helsinki, Finland

**Keywords:** Anaerobic metabolism, Anodic respiration, Mediator-based electron transfer, Lactate dehydrogenase, 2,3-Butanediol

## Abstract

Leveraging alternative electron acceptors to support anaerobic metabolism in industrially relevant microorganisms holds substantial biotechnological potential, especially when coupled to anodic electro-fermentation, which provides a non-depleting electron sink to the microorganisms. *Bacillus subtilis* is a widely used industrial workhorse for biochemical production, valued for its genetic tractability and environmental stress tolerance. However, the anaerobic, anodic metabolism of *B*. *subtilis* has been constrained by limited redox flexibility. Here, deletion of *ldh* (lactate dehydrogenase) to restrict fermentative NAD⁺ regeneration enabled engineered *B. subtilis* to survive anaerobically via anodic respiration, partially oxidising glucose while steering the metabolism toward 2,3-butanediol. The anodic metabolism showed enhanced extracellular electron transfer mediated by ferricyanide, with the highest current density of 0.77 mA/cm^2^ reached within 2 h. Carbon flux was directed predominantly to 2,3-butanediol (0.49 ± 0.07 mol_product_/mol_glucose_) under incomplete glucose oxidation and without pH control. In addition, pH control further improved anodic electro-fermentation performance. At pH 6.5, 66% of the added glucose was consumed, and 2,3-butanediol carbon selectivity rose to 77.1 ± 0.6%, whereas at pH 7.5, cells consumed 89% of the glucose with 73.4 ± 0.7% 2,3-butanediol carbon selectivity. To our knowledge, this is the first investigation of anodic electro-fermentation in *B. subtilis* that integrates metabolic engineering with pH optimisation strategies, demonstrating a new route to produce high-purity biochemicals from renewable resources.

**Supplementary Information:**

The online version contains supplementary material available at 10.1186/s13068-025-02731-5.

## Background

In response to the chemical industry’s persistent dependence on fossil fuels, sustainable bioprocesses powered by industrially relevant microorganisms are being developed. Since its discovery, *Bacillus subtilis* has attracted continuous industrial interest as a versatile microbial cell factory for the production of enzymes, antibiotics and chemicals, owing to its remarkable genetic diversity [1, 2]. For example, *B. subtilis* provides a sustainable microbial route to 2,3-butanediol, a key precursor for solvents, plastics, and synthetic fibres [3]. In addition, its dynamic cell wall architecture and sporulation capacity allow *B*. *subtilis* to survive and propagate under extreme conditions, including outer space [4, 5]. To cope with environmental fluctuations, *B. subtilis* employs multiple adaptive strategies, e.g., enhanced motility, biofilm formation, competence development, membrane remodelling, and anaerobic metabolism using alternative electron acceptors [6–8]. In industrial contexts, exploiting alternative terminal electron acceptors holds great potential in preventing excessive biomass and by-product formation under anaerobiosis [9]. Switching from aerobic to anaerobic operation can improve energetic efficiency and mitigate scale-up limitations associated with oxygen transfer [9–11]. To date, nitrate respiration is the only well-characterised anaerobic respiratory pathway in *B. subtilis*; however, its industrial application is constrained by toxic nitrite (NO_2_^-^) accumulation, reduced biomass constrained by limited energy generation, formation of by-products (e.g., NH_4_^+^, NO, and N_2_O), and complex regulation [8, 12, 13]. Limited anaerobic metabolic flexibility remains a major bottleneck preventing *B. subtilis* from fully grasping its industrial potential [13, 14].

Anodic electro-fermentation (AEF) is a bioelectrochemical process in which certain microorganism respires anaerobically by transferring electrons to a polarised anode electrode [9, 15]. Compared to other forms of anaerobic metabolism, the anode provides a continuously available terminal electron acceptor, allowing cells to remain catalytically active while sustaining efficient synthesis of target products [16]. Recent AEF studies have enhanced conventional fermentations of value-added biochemicals by substituting oxygen with the anode as the terminal electron acceptor with obligate aerobes and facultative anaerobes. Examples include anode-enhanced production of 2-ketogluconic acid by *Pseudomonas putida* [17], lysine by *Corynebacterium glutamicum* [18], acetoin and 2,3-butanediol by *Lactococcus lactis* and *Shewanella oneidensis* [19, 20] and 3-hydroxypropionic acid and 1,3-propanediol by *Klebsiella pneumoniae* [21, 22]. The distinctive role of the anode as an *in-situ* electron sink provides a practical platform to explore and engineer anaerobic metabolism in industrially relevant microorganisms.

Unlike extensively studied exoelectrogens (e.g., *Shewanella oneidensis* and *Geobacter sulfurreducens*), which can directly transfer electrons to solid-state electrodes [23, 24], many industrially relevant gram-positive bacteria, including *B*. *subtilis*, lack native extracellular electron transfer (EET) mechanisms and require engineered pathways and effective electron shuttles [9, 11]. Mediator-based electron transfer involves redox-active chemicals that shuttle electrons between microorganisms and the electrode [25, 31]. *B. subtilis* can utilise ferric iron as a terminal electron acceptor by secreting the siderophore bacillibactin as an iron shuttle and has also been shown to donate electrons to an anode mediated by osmium–redox polymers [26, 27]. Nevertheless, electron transfer capacity has remained limited in prior studies, resulting in rapid cell lysis, comparatively low current densities, and poor substrate conversion rates and yields under strictly anaerobic conditions [11]. The anodic metabolism of *B*. *subtilis* was hypothesised to be hindered either by the slow accumulation of endogenous flavins [28, 29], or by cell envelope barriers that may restrict the accessibility of diffusional mediators [30]. Unlike gram-negative bacteria that possess outer-membrane porins and transporters to facilitate shuttle uptake, *B*. *subtilis* likely lacks such mechanisms, possibly due to its inherently robust peptidoglycan layer and low membrane permeability, which may impede entry of hydrophilic mediators such as ferricyanide. The poor EET compatibility of *B. subtilis* has limited its potential to further develop in AEF systems, necessitating alternative approaches.

A recent study reported that partial inhibition of NAD^+^ regeneration enabled ferricyanide-mediated extracellular electron transfer (EET) and respiration in the gram-positive lactic acid bacterium *L. lactis* [32]. Although not yet fully resolved mechanistically, perturbing the intracellular energy or redox balance of gram-positive bacteria by limiting regeneration of key cofactors (e.g., NAD^+^) may enhance anaerobic capacity under anodic respiration [20, 33]. Most bacteria exhibit sustained NAD^+^ regeneration for glycolysis when transitioning from aerobic to anodic respiration [20, 21]. In *B. subtilis*, the presence of an anode under oxygen-limited conditions facilitated NAD^+^ regeneration and redirected metabolism from lactate toward acetoin production [11], likely as cytoplasmic lactate dehydrogenase encoded by *ldh* is linked to predominantly regeneration of NAD^+^⁺ [13]. However, whether disrupting the primary NAD^+^ regeneration route via *ldh* deletion increases the anaerobic capacity of *B. subtilis* in AEF remains to be determined.

Considering the limitations mentioned, in the present study, we evaluated the potential of *B*. *subtilis* for enhanced anaerobic production of 2,3-butanediol via AEF processes. To perturb the intracellular energy balance, an engineered *B*. *subtilis* strain was prepared by knocking out the gene *ldh*. The mediator-based EET of the engineered *B*. *subtilis* was assessed via two types of AEFs, i.e., anode-assisted (oxygen-limited) and anodic (strictly anaerobic) EFs operated under different pH control strategies. Quantitative metabolite profiling and metabolic flux analysis were used to compare cellular energy/redox balances across these conditions with the goal of increasing 2,3-butanediol yield and selectivity. Finally, the potential mechanisms related to EET of *B. subtilis* were discussed, and implications for further development were outlined.

## Materials and methods

### Strain and medium

*B. subtilis* 168 *trpC*^+^ *xyl*^+^ used in previous studies [5, 11] (hereinafter the parental strain, SI Table S1) was cultivated in LB (per litre containing: 10 g tryptone, 5 g yeast extract, 5 g NaCl) and M9 minimal medium (per litre containing: 8.5 g Na_2_HPO_4_·2H_2_O, 3 g KH_2_PO_4_, 1 g NH_4_Cl, 0.5 g NaCl, 246 mg MgSO_4_·7H_2_O, 0.147 mg CaCl_2_·2H_2_O, 10 mL trace element solution, SI) supplemented with 5 g/L (27.8 mM) glucose as the sole carbon source in all experiments. Agar plates were prepared with the LB medium and 15 g/L agar–agar. Pre-cultures were prepared by picking and transferring single colonies from agar plates into a 14 mL cultivation tube and placed on a rotary shaker (IKA KS4000i Control, Germany) for aerobic overnight cultivation at 300 rpm and 35 °C. Then, 1% (v/v) overnight culture was transferred into baffled shake flasks with fresh LB medium, cultivated under the same conditions. Once the cell density reached OD_600_ = 0.6 (log phase), cells were harvested by centrifugation (at 8000 rpm, 4 °C, 10 min), washed and resuspended in fresh LB medium before transferring into the reactor vessels. An ~ 2.5 mL inoculum was added to each reactor, achieving an initial OD_600_ of 1 in the BES reactor to ensure sufficient cells as biocatalysts under anaerobic conditions [18].

### Construction of *B*. *subtilis* mutant with *ldh* knockouts

To delete the gene encoding lactate dehydrogenase (*ldh*, BSU03050) in *B. subtilis* 168 *trpC*^+^ *xyl*^+^, a linear knockout cassette was constructed using splicing by overlap-extension PCR (SI Tables S1 and S2). The cassette included approximately 850 bp homologous sequences upstream and downstream of the lactate dehydrogenase and a kanamycin resistance gene. The homologous sequences were directly amplified from the genomic DNA of 168 *trpC*^+^ *xyl*^+^. The kanamycin resistance gene, together with a lambda t_0_ terminator, was amplified from plasmid pBAV1K [34]. All primers, PCR reagents and chemicals used in this study were purchased from Merck & Co. (Germany), VWR (USA) and Thermo Fisher Scientific (USA). Detailed information of all primers used in this study can be found in Supplymentary Information.

To prepare the competent cells of *B. subtilis* 168, a single colony from LB–agar plate was inoculated into 5 mL modified Spizizen’s salts medium (per litre containing: 20 g glucose, 1 g casein hydrolysate, 50 mg tryptophan, 11 mg ammonium ferric citrate, 2 g potassium glutamate, 0.36 g/L MgSO_4_, all dissolved in Spizizen’s salts) in a 14 mL cultivation tube at 37 °C and 150 rpm for overnight. Then, 10 mL of fresh modified Spizizen’s salts medium was inoculated with the overnight culture at a 1:50 dilution ratio and incubated under the same conditions. Cell density (OD_600_) was monitored every 30 min, and the culture was harvested 60 min after reaching the end of the exponential growth phase. For transformation, 1 μg of the linear knockout cassette DNA was added to 500 μL of the competent culture and incubated at 37 °C, 150 rpm for 1 h. The transformed cells were then plated directly onto LB–agar plates supplemented with 10 mg/L (17.2 μM) kanamycin sulphate. The resulting strain carried a deletion of *ldh* and was designated as *B. subtilis* 168 *trpC*^+^ *xyl*^+^ Δ*ldh*::*kan*^*R*^ (hereinafter Δ*ldh*).

### Bioelectrochemical systems (BES) setup

Batch bioelectrochemical experiments were conducted using 300 mL H-type borosilicate reactors (Adams and Chittenden Scientific Glass, USA) and a three-electrode setup (SI Fig. S1). The anode and cathode were made of carbon felt (17.9 cm^2^ projected surface area, 1.1 cm thickness, Alfa Aesar, USA) wrapped around a graphite rod (15 cm × 3 mm, Sigma Aldrich, USA), and a platinum wire (10 cm × 0.4 mm, Advent Research Materials Ltd, the UK), respectively. A circular cation exchange membrane (19.6 cm^2^ projected surface area, CMI-7000, Membranes International Inc., USA) was used to separate the two chambers. Anode potential was controlled by a multi-channel potentiostat (VMP3, BioLogic, France) at + 0.5 V versus Ag/AgCl KCl_sat_ reference electrode (SE11NSK7, Xylem, Germany) according to the cyclic voltammetry (CV) analysis of the medium with 1.65 g/L (5 mM) mediator K_3_[Fe(CN)_6_] (SI Fig. S2). In this work, all the potentials are reported against the standard hydrogen electrode (+ 0.197 V vs. Ag/AgCl KCl_sat_).

In advance of anaerobic BES inoculation, anolyte was sparged with N_2_ in the liquid space at a 100 mL/min flow rate using mass flow controllers (EL-FLOW FG, Bronkhorst, the Netherlands) for overnight to ensure the anaerobic condition. After inoculation, N_2_ sparging was switched to the headspace of the reactors at the same flow rate throughout the experiments. For the limited aeration experiment, air was sparged to the liquid phase of the anolyte at a 5 mL/min flow rate using the mass flow controllers. The gas inlet was first attached to sterile vent filters (0.2 µm, 50 mm Millex-FG PTFE, Merck, USA) and to gas washing bottles filled with sterile MQ water, then connected to the reactors. The dissolved oxygen concentration was monitored using O_2_ sensor spots (SP–PSt7–YAU/SP–PSt8–YAU, PreSens, Germany) and a compatible oxygen meter (OXY-1 SMA–BT, PreSens, Germany). Table 1 shows the key AEF parameters tested in this study.

**Table 1:** AEF parameters tested with 168 *trpC*^+^ *xyl*^+^ Δ*ldh* mutant

Type of electro-fermentation (EF)	Operation time (h)	Supplied gas	Redox mediator	Applied electrochemical conditions	pH optimisation
Anode-assisted	286	Air–liquid phase, 5 mL/min	No	+ 0.697 V versus open circuit	No
Anodic	120–244	N_2_-headspace, 100 mL/min	5 mM K_3_[Fe(CN)_6_]	+ 0.697 V versus open circuit	pH controlled to above 6.5 and 7.5

### Analyses

The pH was measured using a pH meter (3110, WTW, Germany) and adjusted using 3 M sterile NaOH when needed. Cell density was analysed photometrically using a spectrophotometer (UV-1800, Shimadzu, Japan) with absorbance at 600 nm wavelength. Each sample was then immediately filtered through 0.2 µm syringe filters (CHROMAFIL^®^ Xtra PET-45/25, Germany) for further analysis. The concentrations of glucose, acetate, formate, succinate, lactate, acetoin and 2,3-butanediol were determined using high-performance liquid chromatography (HPLC) applying a previously developed method [11]. The oxidised mediator (i.e., [Fe(CN)_6_]^3−^) was measured by absorbance at 420 nm and calculated based on the calibration curves made with five concentrations (SI Fig. S3). All equations used for calculations are provided in the Supplementary Information.

### Flux balance analysis (FBA)

The metabolic flux distribution in systems with different pH control strategies was simulated using the COBRApy package [35] in Python with the genome-scale metabolic model iYO844 [36] serving as the computational framework. The model assumed a non-growth condition with maximised glucose uptake rate of 100 mmol/g CDW/h. The production rates for acetate, acetoin, and 2,3-butanediol were constrained using the mean values calculated from three experimentally determined biological replicates, whereas the secretion of all other potential fermentation by-products was prohibited by constraining their exchange fluxes to zero. Any residual carbon not accounted for by the major fermentation products was assumed to enter the tricarboxylic acid (TCA) cycle and be fully oxidised to CO_2_. The native respiratory oxidase reaction was reconfigured to exclude oxygen as the terminal electron acceptor, reflecting the anode as the electron sink in the fermentation system: 2 H^+^ + menaquinol-7 → menaquinone-7 + 4 H^+^. An ATP sink and proton exchange reaction were added to simulate maintenance energy demands and proton dissipation under non-growing conditions.

## Results and discussion

### Anode-assisted metabolism of engineered *B. subtilis*

The Δ*ldh* strain was first confirmed by PCR using primers flanking the *ldh* locus. In addition, phenotypic analysis showed that the strain was unable to grow in M9 minimal medium supplemented with lactate as the sole carbon source, in contrast to the parental strain (168 *trpC*^+^ *xyl*^+^), which exhibited growth under the same conditions, confirming functional disruption of the *ldh* gene. The aerobic growth of the Δ*ldh* on glucose as the sole carbon source compared to its parental strain is presented in Fig. S4 (SI).

**Fig. 1:**
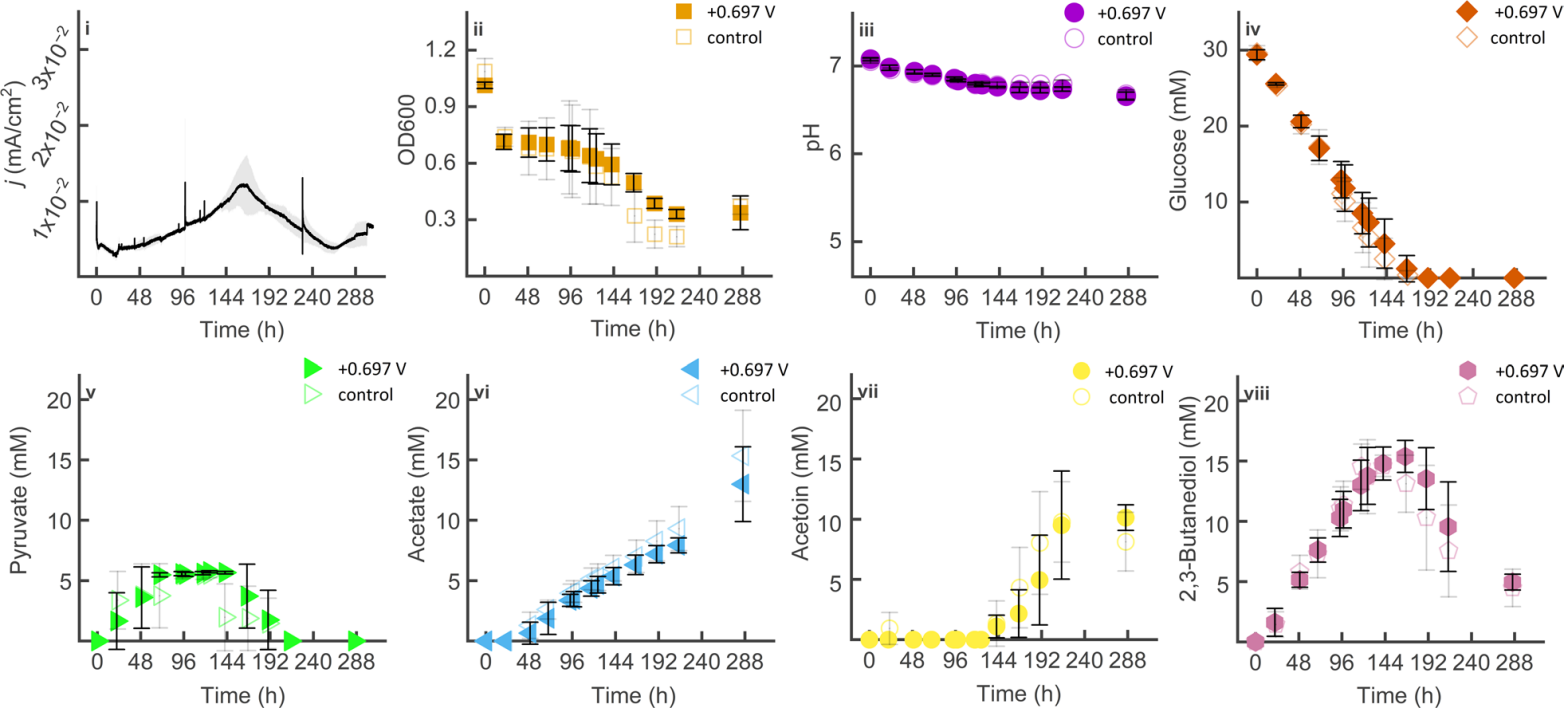
Oxygen-limited cultivation of 168 *trpC*^+^ *xyl*^+^ Δ*ldh* in BES systems under a 5 mL/min aeration rate (anode-assisted EF) using 5 g/L glucose as substrate. Current density was logged at a poised anode potential of + 0.697 V. Current density, optical density, pH, glucose, acetate, pyruvate, acetoin, and 2,3-butanediol concentrations (i–vii) were measured from reactors with applied potential (AP, solid symbols) and open circuit controls (OC, hollow symbols). All results were based on average data of three biological replicates, and standard deviations are represented as coloured areas and error bars

In the previous study, anodic metabolism of the parental strain was hindered under strict anaerobic conditions, resulting in fast cell lysis and poor glucose conversion rates [11]. Meanwhile, the parental strain under anode-assisted EF, i.e., poised anode potential with limited aeration (5 mL/min), showed sustained cell densities with metabolites shifting from lactate (16.3 ± 2.1 mM) to acetoin (17.8 ± 0.6 mM) [11]. Given that the cytoplasmic lactate dehydrogenase is primarily for NAD^+^ regeneration in *B. subtilis* [8], we hypothesised that lactate formation inhibited anodic metabolism by facilitating fermentative NAD^+^ regeneration when oxygen gradually became limited. To support this theory, the Δ*ldh* mutant with the lactate dehydrogenase gene knocked out was tested under the same oxygen-limited condition as before (Fig. 1).

In all reactors under poised potential and open circuit, Δ*ldh* displayed similar readings of planktonic cell densities, pH, glucose and metabolites concentrations (Fig. 1ii–viii). The results first showed pyruvate accumulation from the beginning of the experiment and gradually consumed again after 144 h, accompanied by 2,3-butanediol reassimilation and the start of acetoin formation (Fig. 1v, vii, viii). Without cytoplasmic lactate dehydrogenase to regenerate NAD^+^, the anode was no longer able to steer the metabolism toward acetoin under limited aeration. In contrast, glucose consumption rate dropped from 0.21 ± 0.04 to 0.18 ± 0.03 mM/h with Δ*ldh* compared to the parental strain [11]. Due to catabolite repression, acetate replaced acetoin as the main metabolite with concentrations of 13.0 ± 3.1 mM (+ 0.697 V) and 15.3 ± 3.8 mM (open circuit) by the end of the run (Fig. 1vi, vii). The increased acetate production suggested that when oxygen as an electron acceptor became limited, Δ*ldh* still relied on fermentation activities to maintain the energy balances over using the anode, as acetate fermentation yields additional intracellular ATP in *B. subtilis* [8]. The delayed onset of acetoin production in Δ*ldh* compared to the parental strain (120 h vs. 24 h) [11], suggests a disruption of cellular energy balance and altered redox homeostasis, particularly in NAD^+^/NADH levels, during anode-assisted EF.

Overall, without any additional redox mediator, Δ*ldh* yielded similar low current densities (*j* < 0.02 mA/cm^2^) and coulombic efficiencies (0.48 ± 0.09%) to its parental strain [11] (Fig. 1i; SI Table S3). However, due to the deletion of *ldh* and the lack of effective EET, the anode was unable to outcompete the strong reducing power brought by oxygen. In *B*. *subtilis*, low current densities can be generated from the change of extracellular redox potential [16, 37] or flavin-mediated electron transfer [28]. It has been proposed that flavins released during oxygen-limited metabolism of *B. subtilis* facilitate EET to minerals and electrodes. [25, 28]. However, our results suggested that even with blocked primary fermentative NAD^+^ regeneration, *B. subtilis* still exhibited insufficient electron transfer rates to support effective EF activities with steered metabolites under limited aeration. Further investigation incorporating an additional electron shuttle is warranted to strengthen the electron transfer capacity.

### Extracellular respiration of *Δldh* mediated by ferricyanide via anodic EF

**Fig. 2:**
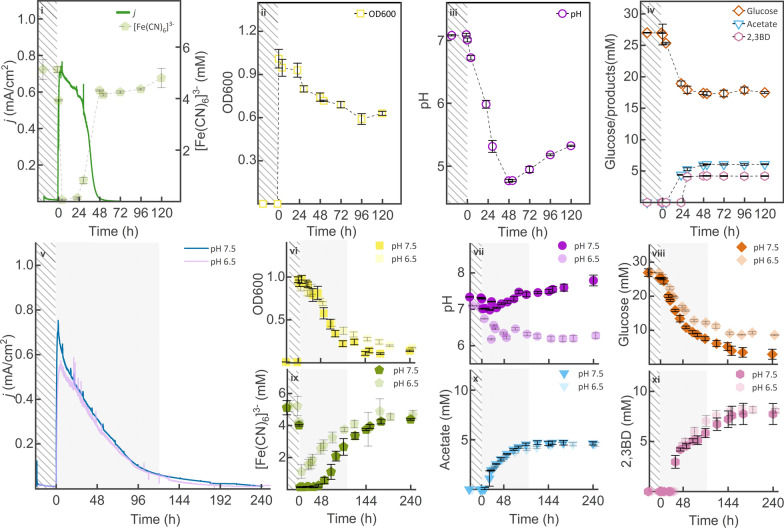
Anaerobic cultivation of 168 *trpC*^+^ *xyl*^+^ Δ*ldh* in BES systems (anodic EF) with 1.65 g/L potassium ferricyanide as redox mediator and 5 g/L glucose as substrate. Current density was logged at a poised anode potential of + 0.697 V. Ferricyanide concentration, optical density, pH, and the concentrations of glucose, acetate, and 2,3-butanediol were measured from reactors without pH controls (hollow symbols, i–iv), and with pH controlled to 6.5 and 7.5 during the initial 100 h, respectively (solid symbols, v–xi). The shaded area marks the period at which the pH was adjusted via the addition of 1 M NaOH at each sampling point, and the shadowed area indicates the period of abiotic experiments. Results from the open-circuit experiments, poised-potential conditions without ferricyanide, and ferrocyanide concentrations across all experiments are provided in the Supplementary Information. All results were based on average data of three biological replicates, and standard deviations are represented as coloured areas and error bars

In AEF, addition of ferricyanide has been reported to facilitate extracellular electron transfer in non-electroactive bacteria [17, 18, 20]. Despite ferricyanide’s low toxicity, increasing its concentration within relatively low ranges (up to 15 mM) have been associated with improved anaerobic biomass growth of certain strains in AEF [10, 20, 38]. Based on toxicity tests with ferricyanide using the parental strain [11], 1.65 g/L (5 mM) ferricyanide was added to the BES reactor. Upon the combination of poised anodic potential and ferricyanide under anaerobic conditions, Δ*ldh* showed immediate and effective anodic respiration with a peak current density of 0.77 mA/cm^2^ and reached in 2 h (Fig. 2i). This value is approximately 35-fold higher than that of the Δ*ldh* under anode-assisted EF and is comparable to the current density reported for ferricyanide-adapted *L*. *lactis* (0.81 ± 0.05 mA/cm^2^) during AEF [20]. In contrast, neither the open-circuit control (with 1.5 mM ferricyanide) nor the poised-potential control (without ferricyanide) using the Δ*ldh* showed measurable glucose oxidation. Moreover, both the Δ*ldh* and its parental strain exhibited rapid cell autolysis in anaerobic BESs [11] (SI Fig. S5). Addition of ferricyanide as redox mediator appeared to be essential for Δ*ldh* to perform anodic respiration without the presence of oxygen, and ferricyanide was turned into the reduced state within 12 h. As the current started to decrease, the mediator was gradually re-oxidised in 48 h with an overall 10.3 ± 0.4 mediator turnover rate. After 28 h, the current production dropped drastically along with decreasing pH and planktonic cell density (Fig. 2ii, iii), while two metabolites, acetate (6.1 ± 0.1 mM), and 2,3-butanediol (4.2 ± 0.1 mM), were detected in the fermentation broth (Fig. 2iv). Neither acetoin nor pyruvate was detected under anodic respiration, in contrast to their presence in the anode-assisted EF. After the current density declined close to zero in 48 h, approximately 35% (9.2 ± 1.7 mM) of total glucose was consumed, while acetate and 2,3-butanediol concentrations remained at the same level. The measured planktonic cell densities continued to drop from (initial) OD 1 to 0.6, indicating that the energy yielded from anodic respiration was insufficient to sustain biomass growth. This limitation was further reflected by incomplete glucose consumption and hindered metabolic activity after 72 h.

### Enhanced anodic respiration with increased 2,3-butanediol production under pH control

The pH showed to be crucial for Δ*ldh* to remain metabolically active under anodic respiration, as an acidic environment (i.e., pH 5.5) inhibited cell metabolism after 48 h (Fig. 2iii, iv). Similar inhibition effects on anodic respiration have been reported in *V. natriegens* and *L. lactis* when pH dropped below 6 [10, 20]. Due to pH drops linked to acetogenic activities, the anode-based metabolism can be further limited by overpotentials and altered redox potential of ferricyanide [10]. In *B. subtilis*, the rapid pH decline during anodic respiration was expected to be contributed by proton accumulation associated with current generation rather than by the formation of acidic products, as acetate was the only acidic metabolite excreted to the fermentation broth with low concentrations (Fig. 2x), and neither of the two control experiments showed any significant change in pH (SI Fig. S5). The M9 minimal medium containing 69.8 mM phosphate buffer offered a buffer capacity of 0.04 M per pH unit at pH 7.2; Meanwhile, the highest concentration of acetate detected (6.1 ± 0.1 mM) theoretically only reduces the pH unit by 0.2. Given that rapid glucose oxidation generates a substantial number of H⁺ during the anodic respiration, limited proton transfer might have occurred across the cation exchange membrane to the cathode chamber, possibly leading to proton accumulation in the anodic chamber and further inhibition of the metabolism [39]. On the other hand, energy imbalance in the Δ*ldh* fermentation pathways likely further limited its anodic respiration by affecting the intracellular pH [8], and the other pH-regulating 2,3 butanediol fermentation pathway was largely restricted under the oxidising power of the anode.

In *B. subtilis*, the lactate and 2,3-butanediol fermentation pathways serve the primary function to maintain cell energy levels by regenerating NAD^+^. In addition, 2,3-butanediol and acetoin fermentation play a secondary role in contributing to pH homeostasis [8]. Under the anodic respiration of Δ*ldh*, we believe that most NAD^+^ were regenerated in 72 h via the electron transfer chain, with the anode serving as the terminal electron acceptor. 2,3-Butanediol as a reduced product, its formation was constrained by the anode’s oxidising potential. With lactate fermentation disabled and 2,3-butanediol synthesis suppressed, the pH-induced metabolic barrier, aggravated by electrochemical reactions, further limited the mediator availability and fermentation activities.

To address the pH-induced metabolic barrier, manual pH control was performed by elevating the pH levels to above 6.5 and 7.5 (± 0.1), respectively (Fig. 2v–xi). Both experiments with pH control exhibited extended current generation up to 240 h, along with increased continuous glucose consumption (16.7 ± 0.7 mM, pH 6.5; 24.0 ± 2.0 mM, pH 7.5), doubled 2,3-butanediol (8.0 ± 0.2 mM, pH 6.5 vs. 7.7 ± 1.0 mM, pH 7.5) and less acetate (4.5 ± 0.2 mM, pH 6.5 vs. 4.6 ± 1.2 mM, pH 7.5) compared to pH-uncontrolled reactors. Maximum current densities in pH 7.5 reactors (0.75 mA/cm^2^) were similar to those without pH control, both surpassing the performance of reactors operated at pH 6.5 (0.57 mA/cm^2^). The highest glucose oxidation took place under pH 7.5, with almost identical levels of acetate and 2,3 butanediol detected compared to pH 6.5. Given that acetate levels remained stable, while current density increased (relative to the pH-uncontrolled condition), energy under anodic respiration was likely generated primarily through proton-gradient-driven ATP synthesis rather than substrate-level phosphorylation. On the other hand, the anode was able to effectively support the regeneration of NAD^+^ from the electron transfer chain, so that other fermentative activities (e.g., acetate and acetoin production) were suppressed.

### Anodic and anode-assisted fermentation of engineered *B. subtilis* for biochemical production

To better understand the electron flow and energy balance of the Δ*ldh* mutant in AEF systems, carbon and redox balances, and yields were determined. The anodic EF results were compared to the anode-assisted EF using Δ*ldh* and the parental strain (Table 2). Under anode-assisted EF, Δ*ldh* displayed slower glucose consumption rates than its parental strain, possibly hindered by the intracellular NAD^+^ level that related to the blocked *ldh* pathway. Under anode-assisted EF, on average, 1.2-fold enhanced acetate and 5.5-fold enhanced 2,3-butanediol yields were obtained in all reactors with applied potential compared to open circuits. However, with the anode no longer influencing the metabolism of the Δ*ldh*, the electron yield dropped by 21-fold compared to the parental strain under similar conditions.

**Table 2:** Calculated yields, glucose consumption rates, and related parameters for Δ*ldh* and parental strain via AEF

	168 *trpC*^+^ *xyl*^+^ [11]		168 *trpC*^+^ *xyl*^+^ Δ*ldh* (this study)				
	Anode-assisted	Anode-assisted, control	Anode-assisted	Anode-assisted, control	Anodic, pH-uncontrolled*	Anodic, pH 6.5*	Anodic, pH 7.5*
Glucose consumption rate (mmol/L/h)	0.31 ± 0.04	0.24 ± 0.05	0.18 ± 0.03	0.19 ± 0.01	0.06 ± 0.01	0.18 ± 0.07	0.19 ± 0.08
Carbon balance (%)	77.60 ± 1.70	80.50 ± 1.72	81.16 ± 2.70	77.74 ± 3.35	84.18 ± 3.65	75.93 ± 0.65	62.71 ± 1.10
Redox balance (%)	89.00 ± 1.73	89.30 ± 2.91	86.36 ± 2.47	85.67 ± 1.74	113.90 ± 5.55	103.89 ± 1.51	94.61 ± 0.81
Yield (mol_product_/mol_glucose_)							
Acetate	0.19 ± 0.06	0.13 ± 0.03	0.22 ± 0.08	0.25 ± 0.09	0.68 ± 0.11	0.29 ± 0.04	0.23 ± 0.05
Acetoin	0.73 ± 0.04	0.56 ± 0.10	0.19 ± 0.12	0.10 ± 0.13	n.d.	n.d.	n.d.
2,3-Butanediol	0.10 ± 0.02	0.23 ± 0.05	0.55 ± 0.10	0.50 ± 0.13	0.49 ± 0.07	0.45 ± 0.02	0.34 ± 0.02
Lactate	0.01 ± 0.01	0.13 ± 0.07	n.d.	n.d.	n.d.	n.d.	n.d.
Electrons	0.002 ± 0.001	n.d.	0.001 ± 0.001	n.d.	5.74 ± 0.98	4.27 ± 0.06	3.74 ± 0.40
2,3-Butanediol selectivity (%)	37.6 ± 2.1	40.0 ± 2.1	57.2 ± 2.5	54.7 ± 2.3	58.1 ± 0.1	77.1 ± 0.6	73.4 ± 0.7

*Incomplete glucose oxidation: calculations were based on the measured glucose consumed. Glucose consumed (% of total added): 35% (pH-uncontrolled), 66% (pH 6.5), and 89% (pH 7.5).

n.d. = not detected.

On the other hand, all three Δ*ldh* anodic respiration experiments at different pHs showed enhanced yields of acetate (ranging from 1.2 to 3.6-fold), 2,3-butanediol (3.4–4.9-fold) and selectivity (58.1–77.1%) compared to the parental strain under anode-assisted EF (37.6% 2,3-butanediol selectivity), with similar trends observed for their production rates (SI Table S3). Controlling pH at 6.5 and 7.5 enhanced total glucose consumed (35% for pH-uncontrolled, 66% for pH 6.5 and 89% for pH 7.5), while the glucose consumption rate (0.18 ± 0.07 mM/h, pH 6.5 vs. 0.19 ± 0.08 mM/h, pH 7.5) was similar to under oxygen-limited anode-assisted EF (0.21 ± 0.04 mM/h for the parental strain and 0.18 ± 0.03 mM/h for Δ*ldh*). Under anodic respiration of Δ*ldh*, electrons became the dominant metabolic output, with the total charge transferred reached 2587 ± 114 C at pH 7.5, 2207 ± 17 C at pH 6.5, and 1489 ± 0 C without pH control (SI Fig. S6). The corresponding coulombic efficiencies (CE) were 8.1 ± 1.1% at pH 7.5, 10.2 ± 0.6% at pH 6.5, and 19.4 ± 2.1% without pH control (SI Table S3), exceeding CE values reported for *V*. *natriegens* (13.67 ± 2.55%) [10], but remaining below that of recombinant *S. oneidensis* SN5 (21.7%) [40]. Under incomplete glucose oxidation, the calculated electron yields were 3.7 ± 0.4 mol_product_/mol_glucose_ at pH 7.5, 4.3 ± 0.1 mol_product_/mol_glucose_ at pH 6.5, and 5.7 ± 1.0 mol_product_/mol_glucose_ under pH-uncontrolled conditions, respectively. At pH 7.5, the redox balance was highest (94.6 ± 0.8%), whereas the carbon balance was lowest (62.7 ± 1.1%), indicating that carbon was diverted to biomass, CO₂, or other undetected by-products. In comparison, the redox balance was 103.9 ± 1.6% at pH-uncontrolled and 113.9 ± 5.6% at pH 6.5; the corresponding carbon balances were 84.2 ± 3.7% and 76.0 ± 1.0%, comparable to anode-assisted EF.

**Fig. 3:**
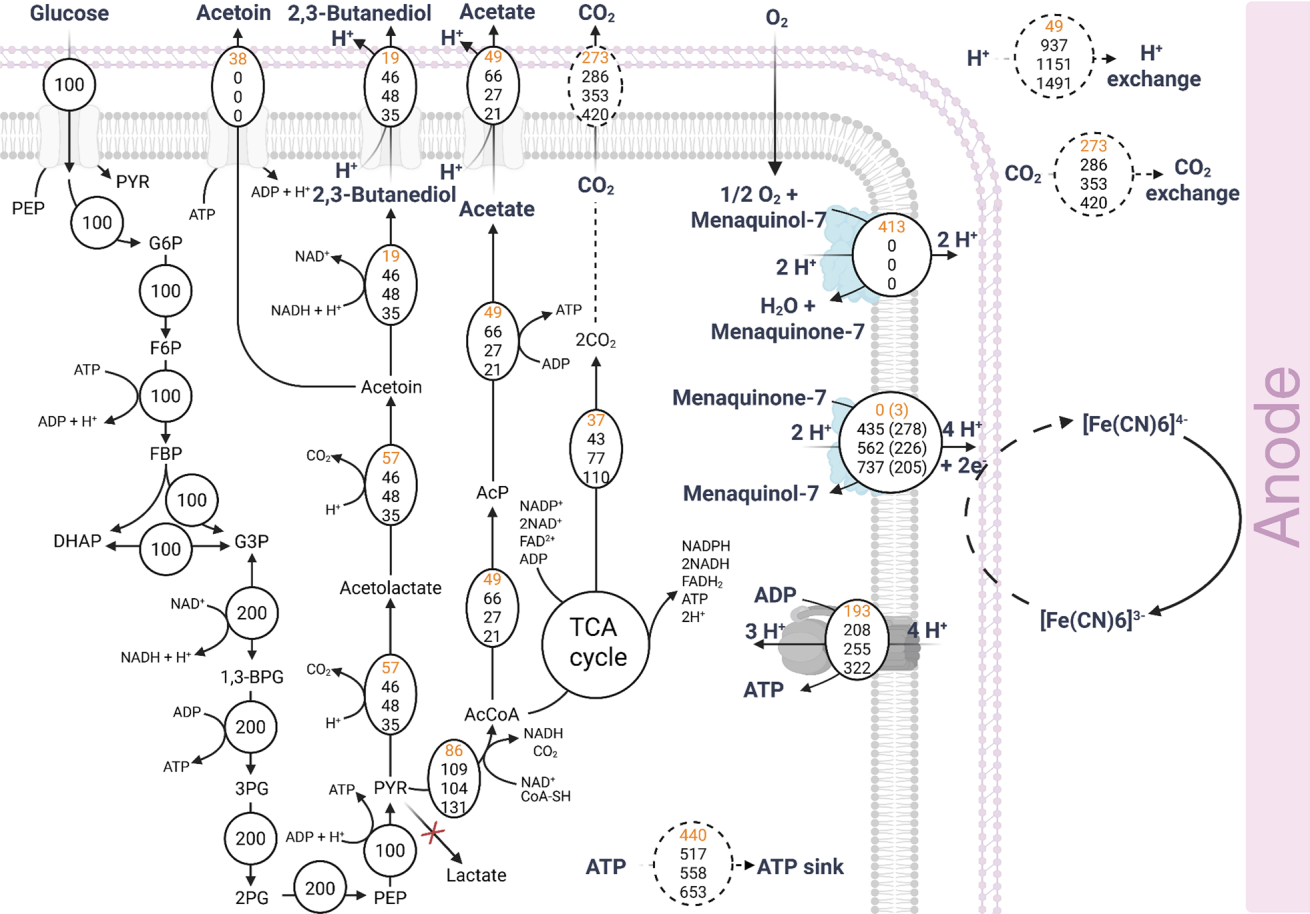
Simulated flux distributions of the 168 *trpC*^+^ *xyl*^+^ Δ*ldh* mutant under anaerobic anodic EF with 5 mM ferricyanide as redox mediator. Solid lines show measured and mass-balance-derived fluxes, and dashed lines indicate assumed fluxes. Simulations represent four conditions, including anode-assisted EF (top numbers), anodic EF with pH-uncontrolled (second numbers), anodic EF with pH controlled to above 6.5 (third numbers), and anodic EF with pH controlled to above 7.5 (bottom numbers), respectively. Identical values across conditions are shown once and experimentally determined electron transfer fluxes normalised to a glucose uptake rate of 100 mmol/g CDW/h are indicated in brackets. Abbreviations: *G6P*, glucose 6-phosphate; *F6P*, fructose 6-phosphate; *FBP*, fructose 1,6-bisphosphate; *DHAP*, dihydroxyacetone phosphate; *G3P*, glyceraldehyde 3-phosphate; *1,3-BPG*, 1,3-bisphosphoglyceric acid; *3PG*, 3-phosphoglyceric acid; *2PG*, 2-phosphoglyceric acid; *PEP*, phosphoenolpyruvic acid; *PYR*, pyruvic acid; *AcCoA*, acetyl-CoA; *AcP*, acetyl phosphate; *ATP*, adenosine triphosphate; *ADP*, adenosine diphosphate; *NAD*^+^*/NADH*, nicotinamide adenine dinucleotide (oxidised/reduced); *NADP*^+^*/NADPH*, nicotinamide adenine dinucleotide phosphate (oxidised/reduced)

Based on the measured metabolites and electrons, flux balance analysis was performed to compare operation strategies for enhancing electron and 2,3-butanediol production with Δ*ldh*. (Fig. 3). For simplicity, we hereby propose that ferricyanide has facilitated extra-cytoplasmic electron transfer under anodic respiration, likely directly or indirectly through the inner membrane menaquinone (MK) pool, i.e., via the oxidation of menaquinol-7 to menaquinone-7 [8]. The MK pool is known as the central membrane-embedded quinone electron carrier that channels electrons from NADH dehydrogenase through the respiratory chain to terminal electron acceptors [27, 41]. In both pH-controlled systems, FBA exhibited greater electron transfer to the anode, higher CO_2_ production, and increased ATP formation compared with pH-uncontrolled anode-assisted and anodic EF (Fig. 3). However, the actual electron transfer fluxes captured by the anode in pH-controlled systems (values in brackets) were much lower than the FBA simulation results. Given the lower carbon and redox balances calculated under increased glucose consumption in pH-controlled systems (Table 2), the unaccounted carbon was likely redirected into other undetected by-products rather than being released as CO_2_. Due to the Δ*ldh* harbouring *kan* and the addition of kanamycin as a selection marker, cells may require additional energy to inactivate kanamycin by bearing the metabolic burden of kanamycin kinase and managing associated stress responses [42]. Therefore, an overall decreased energy yield for the growth of Δ*ldh* was expected, yet cells remained metabolically active in the pH-controlled systems compared to those without pH controls. Since real-time biofilm measurement was not feasible in the BES, biofilm-related activities may also have contributed to the unaccounted energy and carbon observed in the FBA. Overall, pH 6.5 favoured higher flux toward 2,3-butanediol, whereas at pH 7.5 cells remained metabolically active for a longer period under anodic respiration, with a higher glucose consumption rate compared with pH 6.5.

### Limitations of engineered *B. subtilis* EET and outlook for anodic respiration

Given the hydrophilic and highly charged nature of ferricyanide, it remains unclear whether this trianionic mediator can reach redox-active sites in *B. subtilis* across its robust (up to 5000 disaccharide units), anionic peptidoglycan-teichoic acid matrix [30]. Electrostatic repulsion by wall teichoic acids in gram-positive envelopes may exclude ferricyanide from approaching membrane-embedded electron-transfer components [43, 44]. On the other hand, Coman and colleagues demonstrated interfacial, polymer-wired electron transfer in *B. subtilis* that does not require penetration of the cytoplasmic membrane, and suggested that succinate/quinone oxidoreductase coupled extracellular current from the respiratory chain [27]. Assuming EET occurs external to the cytoplasmic membrane, even if ferricyanide traverses the *B. subtilis* peptidoglycan matrix, electron transfer may still be limited by insufficient NADH/quinol supply to the membrane quinone pool and/or by premature re-oxidation or quenching of secreted electroactive compounds (e.g., flavins) before they encounter ferricyanide, thereby reducing EET efficiency, similar to observed previously [11]. In the case of anodic respiration of Δ*ldh*, suppressing the fermentative NAD^+^ regeneration route may liberate additional NADH for quinone reduction, thereby enhancing extracellular electron transfer under anodic respiration. However, under anodic respiration, Δ*ldh* exhibited no observable growth, conceivably due to ferricyanide toxicity, antibiotic burden from kanamycin selection, and/or a redox-energy imbalance of the engineered strain. Consequently, only the initial inoculum likely remained metabolically active, limiting glucose oxidation and thereby constraining overall AEF performance. Overall, the mechanisms of the engineered *B*. *subtilis* EET need to be closely investigated to further improve the substrate consumption with enhanced product yields or electron flux to the anode.

## Conclusions

In summary, deletion of *ldh* in *B. subtilis* enhanced its EET capacity. By restricting fermentative NAD^+^ regeneration and perturbing intracellular redox and energy balance, the engineered *B*. *subtilis* strain facilitated ferricyanide-mediated electron transfer to the anode. Fermentative NAD^+^ regeneration emerged as a key determinant of *B. subtilis* extracellular respiration and anodic metabolism. Relative to oxygen-limited anode-assisted EF, strictly anodic respiration delivered ~ 35-fold higher current density and a comparable 2,3-butanediol yield of 0.49 ± 0.07 mol_product_/mol_glucose_, with an enhanced carbon selectivity of 58.1 ± 0.1%. In addition, pH control further tuned the AEF performance. At pH 6.5, 2,3-butanediol selectivity increased to 77.1 ± 0.6%, whereas at pH 7.5, total charge transferred to the anode was maximised (2587 ± 114 C). Combined with flux balance analysis, anodic respiration indicated ATP generation relied primarily on chemiosmosis rather than substrate-level phosphorylation. Together, these results demonstrated that straightforward metabolic engineering enabled anaerobic anodic respiration in an otherwise oxygen-dependent gram-positive bacterium, and when combined with pH control strategies, AEF empowered high-yield, high-selectivity production of reduced biochemicals.

## Supplementary Information

Additional file 1.

## Data Availability

All data supporting the findings of this study are available within the paper and its Supplementary Information.
