# Supplementary material for: Enhanced extracellular respiration of engineered Bacillus subtilis via anodic electro-fermentation with pH optimisation
Source: Biotechnol Biofuels Bioprod. 2026 Jan 3;19:8. doi: 10.1186/s13068-025-02731-5 (PMC12853675; doi:10.1186/s13068-025-02731-5)
Supplement: Supplementary file 1 — Additional file 1. [file 13068_2025_2731_MOESM1_ESM.pdf]

Supplementary Information

Enhanced extracellular respiration of engineered *Bacillus subtilis* via anodic electro-fermentation with pH optimisation

Yu Sun [a,*] Changshuo Liu [a], Igor Vassilev [a], Antti J. Rissanen [a,b], Jin Luo [a] and Marika Kokko [a]

[a] Faculty of Engineering and Natural Sciences, Tampere University, Korkeakoulunkatu 8, 33720 Tampere, Finland

[b] Natural Resources Institute Finland, Latokartanonkaari 9, 00790, Helsinki, Finland

[*] Corresponding author

E-mail addresses: yu.sun@tuni.fi (Y. Sun)

ORCID: orcid.org/0000-0002-0343-7393 (Y. Sun)

The trace element solution containing per litre: 1.5 mg EDTA, 450 mg $ZnSO_4 \cdot 7H_2O$, 30 mg $CoCl_2 \cdot 6H_2O$, 100 mg $MnCl_2 \cdot 4H_2O$, 30 mg $CuSO_4 \cdot 5H_2O$, 1.4 g $FeSO_4 \cdot 7H_2O$, 40 mg $Na_2MoO_4 \cdot 2H_2O$, 100 mg H_3BO_3 and 10 mg KI.

The mediator turnover rate (TTN) was calculated using the equation described by Gemünde et al. (2023):

$$TTN = \frac{e_{anode}(t)}{m_{mediator} \cdot z_{mediator}} \tag{1}$$

where e_{anode}(t) denotes the quantity of total transferred electrons (mmol) recorded at the anode at time t, $m_{mediator}$ is the absolute amount of ferricyanide in the system, $z_{mediator}$ is the number of electrons transferred per mediator turnover (for ferricyanide, $z_{mediator} = 1$).

The quantity of transferred electrons to the anode during the electro-fermentation were quantified by integrating the recorded current over time (1 A·s = 1 C) and converting charge to molar electrons using Faraday's constant (F = 96485.33 C/mol). Data of all time points were added to give the total mol of electrons, with glucose concentrations and the reactor volume incorporated into the calculations. The average current densities were determined from the total charge transferred (Q_{anode}) normalised to the electrode's projected surface area (17.9 cm^2) and operation time. Product yield coefficients were calculated as the slope of plots of moles product versus total moles of glucose consumed.

The carbon balance (CB) was calculated for each measuring point using the equation below:

$$CB\ (\%) = \frac{[\sum_i (m_i \cdot n_i)_t]}{[\sum_i (m_i \cdot n_i)_{t_0}]} \times 100 \quad (2)$$

where m_i is the absolute amount (mmol) of compound i at a specific time t; n_i is the number of carbon atoms of compound i. Time t_0 denotes the point of inoculation.

The redox balance (RB) was calculated for each measuring point using the equation below:

$$RB\ (\%) = \frac{[\sum_i (m_i \cdot n_i \cdot \gamma)_t + e_{anode}(t)]}{[\sum_i (m_i \cdot n_i \cdot \gamma)_{t_0} + e_{anode}(t_0)]} \times 100 \quad (3)$$

where $e_{anode}(t)$ is the mmol of electrons transferred to the anode at specific time t and t_0 initial 0 h. The carbon and redox balance calculations did not consider any biomass and gas production (e.g., CO_2 and H_2) due to the continuous air or N_2 sparging in all systems.

The degree of reduction (γ) of the respective chemical with the elemental composition $C_aH_bO_cN_d$ was calculated based on the equation below:

$$\gamma = \frac{a \times 4 + b \times 1 + c \times (-2) + d \times (-3)}{a} \quad (4)$$

The carbon selectivity (CS) was calculated based on the equation below:

$$S_i^{Carbon}(\%) = \frac{n_{Carbon,i}\ c_i}{\sum_j n_{Carbon,j}\ c_j} \quad (5)$$

where c_i is the molar concentration of product i (mol/L) and $n_{Carbon,i}$ is the number of carbon atoms per molecule of i. The denominator sums across all quantified products j included in the carbon pool.

The coulombic efficiency (CE) was calculated based on the equation below:

$$CE\ (\%) = \frac{Q_{anode}}{Q_{(glucose\ consumed)} - Q_{metabolites}} \times 100 \quad (6)$$

where Q_{anode} is the total charge transferred to the anode during the experiment. $Q_{(glucose\ consumed)}$ and $Q_{metabolites}$ are charge contained in the consumed glucose and produced metabolites in the form of reducing equivalents.

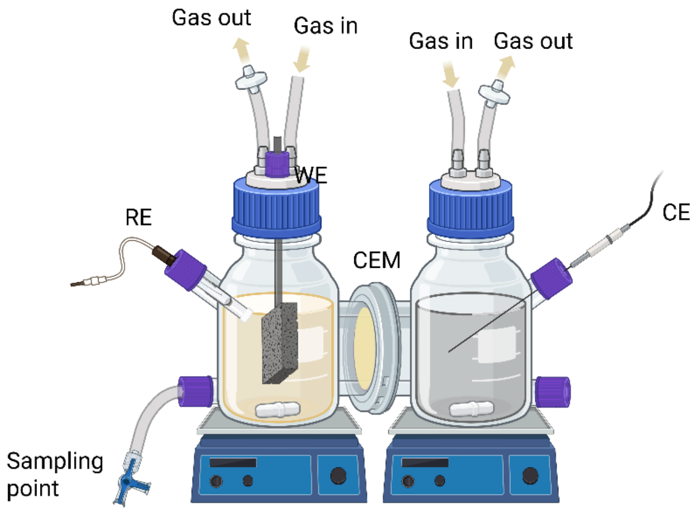

Fig S1. Schematic overview of bioelectrochemical reactors used in this study. CEM: cation exchange membrane; WE: working electrode; RE: reference electrode; CE: counter electrode. Created with BioRender.com.

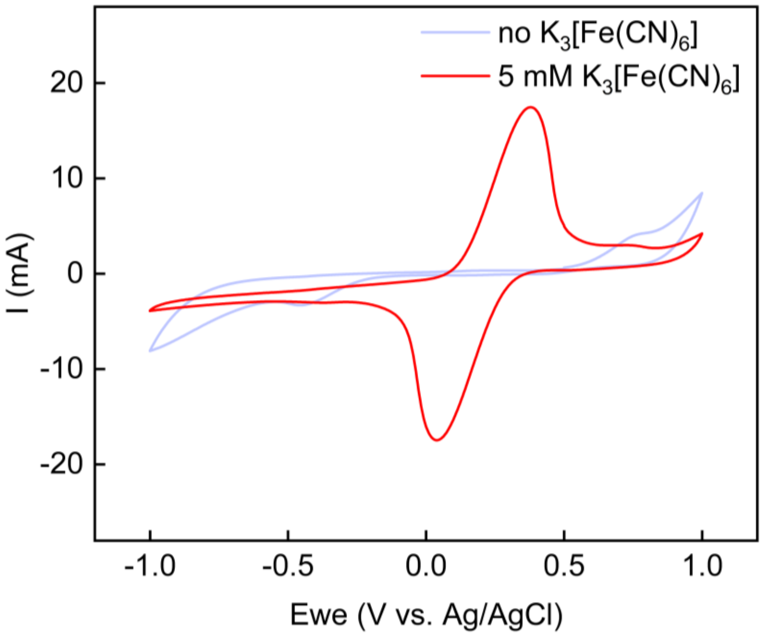

Fig S2. Abiotic electrochemical characterisation of graphite felt and carbon rod combination as the working electrode and 5 mM $K_3[Fe(CN)_6]$ via cyclic voltammetry at a scan rate of 0.10 mV/s within a potential window from 1.0 to -1.0 V vs. Ag/AgCl KCl_{sat}. The results were the second cycle out of three cycles scanned.

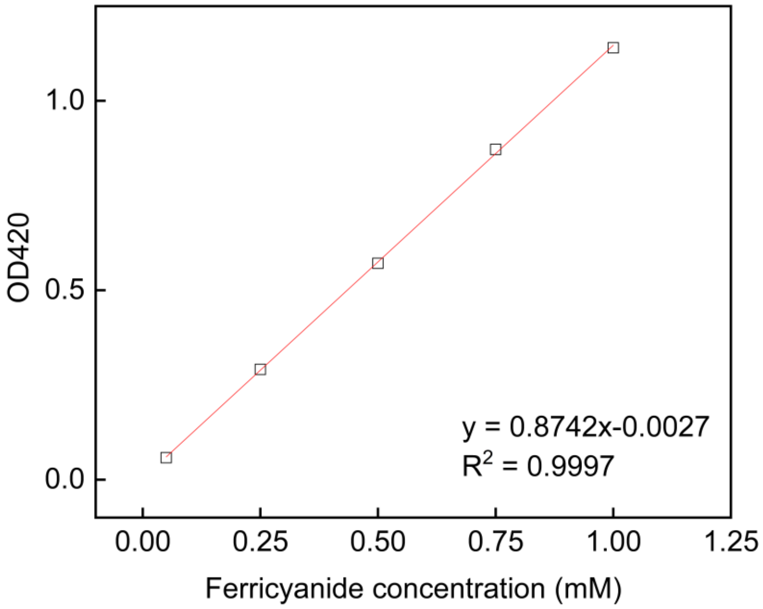

Fig S3. Calibration of the oxidised mediator (ferricyanide). Five dilutions prepared from a 1 mM $K_3[Fe(CN)_6]$ stock were measured at OD 420nm, and absorbance was plotted versus concentration. The red line shows the linear fit of the 7 data points.

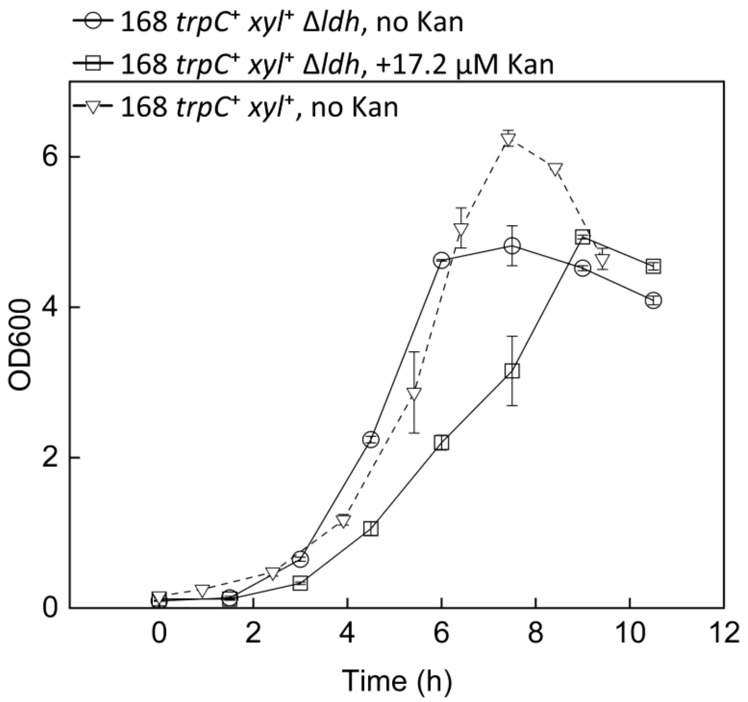

Fig S4. Growth of *B. subtilis* 168 *trpC*+ *xyl*+ and 168 *trpC*+ *xyl*+ Δ*ldh* in aerobic shake flasks in M9 minimal medium supplemented with 5 g/L glucose, with or without kanamycin sulphate (17.2 μM). Each curve shows the mean of three to four independent biological replicates and standard deviations are represented as error bars.

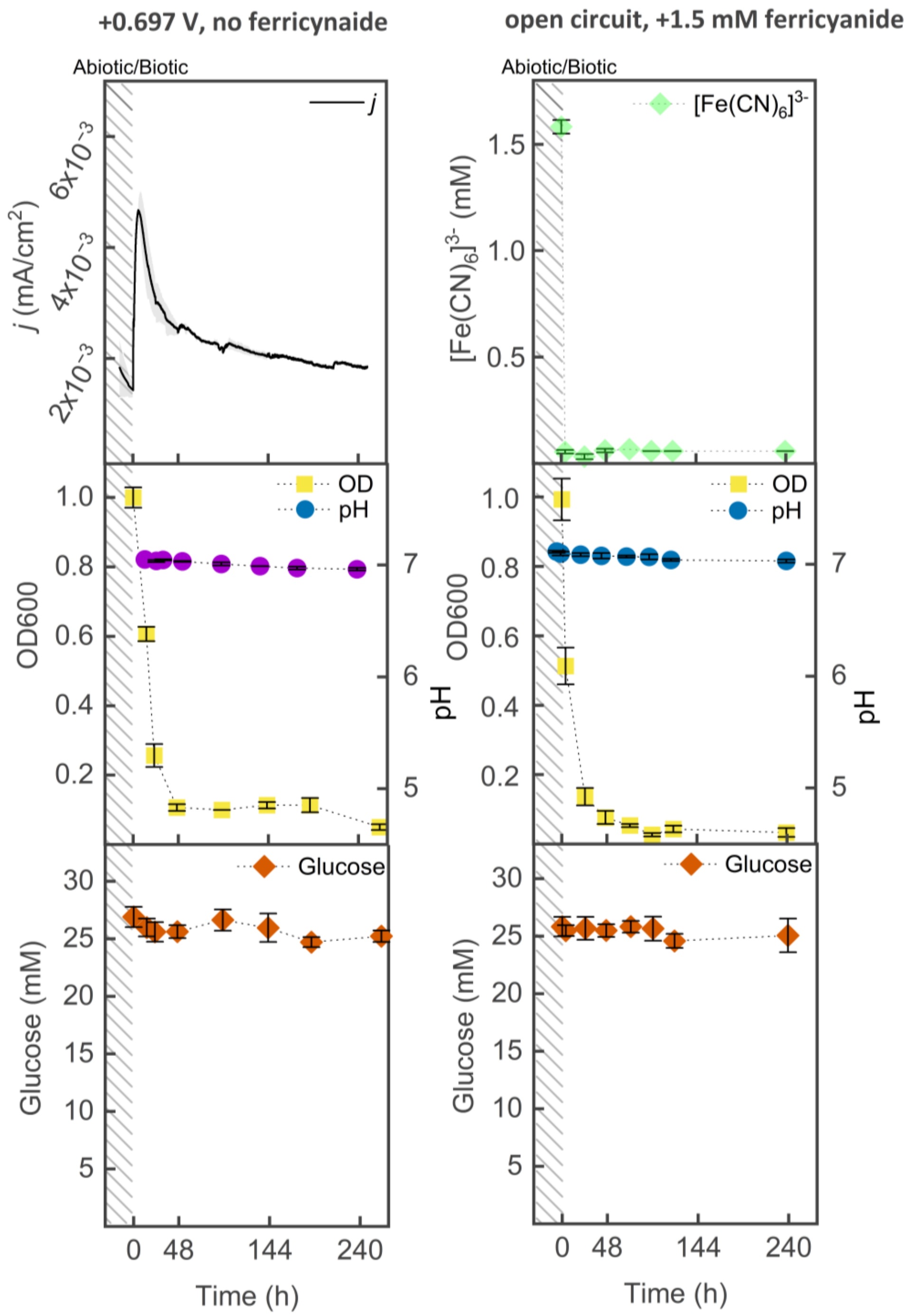

Fig S5. Anaerobic cultivation of 168 *trpC⁺ xyl⁺ Δldh* in BES systems without ferricyanide at +0.697 V and with addition of 1.5 mM ferricyanide without poised potential (open circuit). Results are the mean of three to four independent biological replicates and standard deviations are represented as coloured areas and error bars. The shaded area indicates the period of abiotic experiments.

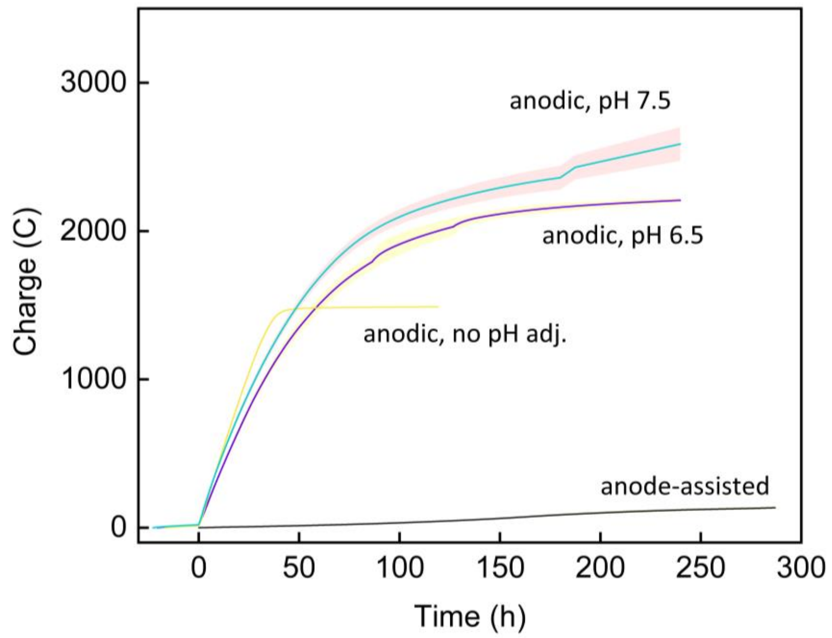

Fig S6. Total charge transferred (C) under anode-assisted EF and anodic EF of *B. subtilis* 168 *trpC*$^+$ *xyl*$^+$ Δ*ldh* at a poised anode potential of +0.697 V vs Ag/AgCl KCl_{sat}.

Table S1. *Bacillus subtilis* strains used in this study

**Strain**	**Description**	**Origin/BGSC catalogue number**
168	*B. subtilis* subsp. subtilis, common laboratory strain, auxotrophic (*trpC2*)	1A1
NCIB 3610^T	Putative parent strain of *B. subtilis* 168, prototrophic	3A1
168 *trpC*$^+$	168 with *trpC* of NCIB 3610^T	Averesch & Rothschild (2019)
168 *trpC*$^+$ *xyl*$^+$	168 with *trpC* of NCIB 3610^T, xylose adapted	Averesch & Rothschild (2019)
168 *trpC*$^+$ *xyl*$^+$ Δ*ldh*	168 $trpC_{NCIB3610}$, xylose adapted, Δ*ldh*::*kan*^{R}	This study

Table S2. Primers used in this study

ID	Sequence (5'-3')	Description
BS_1	aaagctggagcgggttcatt	Amplification of the upstream homologous sequence of *ldh*.
BS_2	catccttccagggtatgtttct	
BS_3	agtcaactaaccgcaactttagag	Amplification of the downstream homologous sequence of *ldh*.
BS_4	tgccatttgcggaggaagag	
BS_5	aagagaaacataccctggaaggatgttt atgatttcctctagaagcggc	Amplification of the kanamycin resistance gene, with 25 bp overlapping sequences homologous to the homologous sequences upstream and downstream of *ldh*.
BS_6	actctaaagttgcggttagttgacttcgc ctagtgcttggattct	
BS_7	aaatgcaggttcatcctctg	Verification of *ldh* knockout
BS_8	tttgctgatgcagaaaaggc	Verification of *ldh* knockout

0 **Table S3.** Calculated production rates for Δ*ldh* mutant and parental strain via AEF.

	168 *trpC*[+] *xyl*[+] [3]		168 *trpC*[+] *xyl*[+] Δ*ldh* (this study)				
	**anode-assisted**	**anode-assisted, control**	**anode-assisted**	**anode-assisted, control**	**anodic, pH-uncontrolled***	**anodic, pH 6.5***	**anodic, pH 7.5***
Production rate (mmol/L/h)							
Acetate	0.03 ± 0.01	0.02 ± 0.01	0.03 ± 0.01	0.04 ± 0.01	0.05 ± 0.01	0.05 ± 0.02	0.05 ± 0.02
Acetoin	0.12 ± 0.02	0.08 ± 0.02	0.03 ± 0.01	0.03 ± 0.01	n.d.	n.d.	n.d.
2,3-butanediol	0.20 ± 0.04	0.13 ± 0.04	0.09 ± 0.02	0.09 ± 0.03	0.04 ± 0.01	0.07 ± 0.02	0.06 ± 0.02
Lactate	0.29 ± 0.06	0.25 ± 0.04	n.d.	n.d.	n.d.	n.d.	n.d.
Electrons	$(2.30 \pm 0.90) \times 10^{-5}$	n.d.	$(3.00 \pm 2.00) \times 10^{-5}$	n.d.	0.43 ± 0.01	0.33 ± 0.01	0.38 ± 0.03
Coulombic efficiency (%)	0.51 ± 0.14	n.d.	0.48 ± 0.09	n.d.	19.42 ± 2.07	10.16 ± 0.55	8.14 ± 1.11

n.d. = not detected

*Incomplete glucose oxidation: calculations were based on the measured glucose consumed. Glucose consumed (% of total added): 35% (pH-uncontrolled), 66% (pH 6.5), 89% (pH 7.5).

Sequence of the lactate dehydrogenase knockout cassette.

aaagctggagcgggttcatttcaagtgaacgatggtaaactgacaggcacgatcaatgccaggtctgtagctgtgctttatcctgatgat
attgcaaaagcgcctcatgttttccttgagaattacaaaacaggtgtaacacattctttcaatgatcaactgacgattaccttgcgtgcagat
gcgaatacaacaaaagccgtttatcaaatcaataatggaccagagacggcgtttaaggatggagatcaattcacaatcggaaaaggag
atccatttggcaaaacatacaccatcatgttaaaaggaacgaacagtgatggtgtaacgaggaccgagaaatacagttttgttaaaaga
gatccagcgtcggccaaaaccatcggctatcaaaatccgaatcattggagccaggtaaatgcttatatctataaacatgatgggagccg
agtaattgaattgaccggatcttggcctggaaaaccaatgactaaaaatgcagacggaatttacacgctgacgctgcctgcggacacg
gatacaaccaacgcaaaagtgatttttaataatggcagcgcccaagtgcccggtcagaatcagcctggctttgattacgtgctaaatggt
ttatataatgactcgggcttaagcggttctcttccccattgagggcaaggctagacgggacttaccgaaagaaaccatcaatgatggttt
ctttttgttcataaatcagacaaaacttttctcttgcaaaagtttgtgaagtgttgcacaatataaatgtgaaatacttcacaaacaaaaaga
catcaaagagaaacataccctggaaggatgtttatgatttcctctagaagcggccgcgaattcgacgtcaaattctatcataattgtggttt
caaaatcggctccgtcgatactatgttatacgccaactttgaaaacaactttgaaaaggctgttttctgtatttaaggttttagaatgcaagg
aacagtgaattggagttcgtcttgttataattagcttcttggggtatctttaaatactgtagaaaagaggaaggaaataataaatggctaaa
atgagaatatcaccggaattgaaaaaactgatcgaagaataccgctgcgtaaaagatacggaaggaatgtctcctgctaaggtatataa
gctggtgggagaaaatgaaatcctatatttaaaaatgacggacagccggtataaagggaccacctatgatgtggaacgggaaaagga
catgatgctatggctggaaggaaagctgcctgttccaaaggtcctgcactttgaacggcatgatggctggagcaatctgctcatgagtg
aggccgatggcgtcctttgctcggaagggtatgaagatgaacaaagccctgaaaagactatcgagctgtatgcggagtgcatcaggc
tctttcactccatcgacatatcggattgtccctatacgaatagcttagacggccgcttagccgaattggattacttactgaataacgatctg
gccgatgtggattgcgaaaactgggaagaagacactccatttaaagatccgcgcgagctgtatgatttttttaaagacggaaaagcccg
aagaggaacttgtcttttcccacggcgacctgggagacagcaacatctttgtgaaagatggcaaagtaagtggctttattgatcttggga
gaagcggcagggcggacaagtggtatgacattgccttctgcgtccggtcgatcagggaggatatcggggaagaacagtatgtcgag
ctatttttgacttactggggatcaagcctgattgggagaaaataaaatattatattttactggatgaattgttttagtaccgagctcgcttgga
ctcctgttgatagatccagtaatgacctcagaactccatctggatttgttcagaacgctcggttgccgccgggcgttttttattggtgagaat
ccaagcactaggcgaagtcaactaaccgcaactttagagtaaagggctgattgtcaatgtgggagcagttgtatgatccgtttggaaac
gagtatgtgagcgcacttgtggcgctcactccgattctctttttcttttggctttaactgttttgaaaatgaaaggcattcttgcggcatttctt
accctagccgtcagtttcttcgtctccgtttgggcatttcatatgccggttgaaaaagcgatttcttctgttttgttaggaatcgggagcggg
ctgtggcccattggctacatcgtcctgatggcggtgtggctgtataaaatcgccgtgaaaaccgggaaatttaccattattcggtccagc
attgccggcatttcgcctgaccaacgattacagctattattaattggtttttgttttaacgcgtttttagaaggcgcggccggttttggtgttcc
gattgcgattagtgcggcgctgctcgtcgaacttggttttaaaccgttaaaagcggcggcgctctgcttgattgcaaacgctgcctccgg
agcctttggggcgattgggattcctgtcatcacaggggcgcagattggtgatttgtctgctcttgagctgtctcggacattaatgtggaca
ctgccgatgatctcatttttaataccattcctgcttgtattcttattagaccgaatgaaaggaatcaaacagacatggcccgctcttctggttg
tgagcggtgggtatacagcggttcagacactgacaatggcggtgctcgggccggaattagcaaacattttggcggccttattcagcat
gggcgggcttgccctcttcctccgcaaatggca

References

1. Gemünde A, Gail J, Holtmann D. Anodic Respiration of Vibrio natriegens in a Bioelectrochemical System. ChemSusChem. 2023;16:e202300181. https://doi.org/10.1002/cssc.202300181

2. Averesch NJH, Rothschild LJ. Metabolic engineering of *Bacillus subtilis* for production of *para* -aminobenzoic acid – unexpected importance of carbon source is an advantage for space application. Microbial Biotechnology. 2019;12:703–14. https://doi.org/10.1111/1751-7915.13403

3. Sun Y, Kokko M, Vassilev I. Anode-assisted electro-fermentation with Bacillus subtilis under oxygen-limited conditions. Biotechnology for Biofuels and Bioproducts. 2023;16:6. https://doi.org/10.1186/s13068-022-02253-4
